# Views of Mental Health Professionals on Positive Changes in Service Practices and Staff-User Relationships After One Year of Covid-19 Pandemic in Italy

**DOI:** 10.1007/s40737-022-00259-7

**Published:** 2022-01-20

**Authors:** Lorenza Magliano, Tommaso Bonavigo, Claudia Battiston, Alessandra Oretti, Roberta Accardo, Gabriella D’Ambrosi, Gaetana Affuso, Elisabetta Pascolo-Fabrici

**Affiliations:** 1 Department of Psychology, University of Campania “Luigi Vanvitelli”, Viale Ellittico 31, 81100 Caserta, Italy; 2 Department of Mental Health, Giuliano Isontina University Health Authority (Azienda Sanitaria Universitaria Giuliano Isontina - ASUGI), Trieste, Italy; 3 Clinical Department of Medical, Surgical and Health Sciences, University of Trieste, 34149 Trieste, Italy

**Keywords:** COVID-19, Mental health staff, Mental health services, Service users, Staff views

## Abstract

This study explored views of mental health services (MHS) professionals regarding positive changes in service practices and organizations, and staff-user relationships after one year of COVID-19 in Italy. Professionals from a community-oriented MHS completed online the Questionnaire on MHS Transformations during the COVID-19 pandemic, a 30-item tool developed by a participatory approach and validated. Of the 184 participants, 91.8% felt it was “true/definitely true” that during the pandemic they had informed users on procedures to reduce contagion risks, and 82.1% stated that they had increased telephone contact with users. Sixty-nine percent of professionals reported that staff revised treatment plans according to new needs of care and 78.6% stated that they had been able to mediate between user needs and safe working procedures. Moreover, 79.4% of respondents stated that they had rediscovered the importance of gestures and habits, and 65% that they had gained strength among colleagues to face fear. Fifty-four percent of participants admitted that they had discovered unexpected personal resources in users. Overall, 59.6% of participants stated that they found some positives in the COVID-19 experience. Perceived positive changes was greater among professionals from community facilities vs. those from hospital and residential facilities. In community-oriented MHS, the pandemic offered an opportunity to change practices and rethink the meaning of relationships between people. This data may be useful in generating a more balanced understanding of COVID-19's impact on MHS and for MHS planning in the pandemic era.

## Introduction

Since the early phase of COVID-19 pandemic, research has shown an increase of mental health problems in the general population [23, 30] and negative effects on mental health services (MHS) [12, 30], particularly in the reduction of hospital and community activities, and the closure of residential facilities [2, 5, 26]. In persons with mental disorders, clinical worsening, increased social isolation [9], and difficulties in accessing care were reported [26]. Among the staff, stress and burnout were indicated [22], mainly related to abrupt work changes, fear of contagion, and difficulty in ensuring acceptable levels of care to persons with severe mental disorders [15].

While negative effects of COVID-19 pandemic on MHS have been largely described, its potential beneficial effects have been relatively unexplored. The preponderance of difficulty-focused studies [4, 9, 15, 26] may have led to an underestimation of the role those adaptive mechanisms may have exerted on MHS staff, positively influencing relationships and practices. Available data revealed that sometimes the COVID-19 pandemic has led to increased staff collaboration, reduced bureaucracy, and greater flexibility in healthcare [1, 11, 15]. Remote work was useful to facilitate staff participation in meetings and to provide psychological support and clinical assessments to users [4, 15, 26]. Staff perception of the pandemic as leading something useful was also reported [4]. In users with severe mental disorders, the widespread reduction in social relationships has been found to be associated with reduced marginalization and increased empathy toward them [9]. The focus on the pandemic seemed to distract some users from their mental condition, apparently leading to symptom relief [26] and a greater community involvement [9]. Available studies were often conducted using qualitative methodologies, which are difficult to adopt in routine settings, or through poorly validated tools. Moreover, most studies were conducted at the beginning of the pandemic, when coping mechanisms were centered on initial emotional reactions, but these were later replaced by problem solving strategies [17]. It is likely that the persistence of COVID-19 pandemic has led mental health professionals to develop new organizational and intervention approaches, and to give new values to their work and the relationships with users and their families. Knowledge of this data may be useful in generating a more balanced understanding of COVID-19 pandemic's impact on MHS and for mental health policy planning in the pandemic era [10].

During the first year of the COVID-19 pandemic, Italy was one of the most affected European countries, with 2,800,000 infected people and 95,000 fatalities at one year [14, 21]. During the first lockdown (from March 9 to May 18, 2020), MHS activities dropped by 30% overall, while voluntary and compulsory hospital admissions dropped by 50% and 70%, respectively [2, 24]. In MHS closely aligned to a community-oriented approach, outpatients services continued to operate normally during the emergency, while hospital services decreased their activities [3]. As of summer 2020, most clinical and rehabilitation activities gradually resumed at the pre-COVID-19 levels, including collaboration of the third sector and user and family associations [7].

This study explored views of MHS professionals regarding the beneficial changes occurred in service practices and organizations, and in staff-user relationships during the first year of the COVID-19 pandemic in Italy. The research was conducted in the Trieste MHS, one of the MHS network with the longest experience of community care in Italy and internationally [20, 28]. The study was carried out using an ad hoc questionnaire developed by a participatory approach and then validated. In particular, the study examined views of the MHS staff regarding the following issues:

- Acknowledgement of user capabilities
- Awareness and value of teamwork
- Flexibility and ability to reinvent the service
- Maintenance and introduction of best practices
- Acknowledgement of positive aspects in the pandemic experience.

With respect to the issues listed above, the study tested whether the perception of beneficial changes was greater in staff from community services vs. those from hospital and residential services.

## Materials and Methods

### Study Design and Participants

The research was conducted in the Trieste MHS from September to December 2020 (preliminary phase of instrument development) and from February to March 2021 (main phase of data collection). The Trieste MHS is managed by the Department of Mental Health of the Giuliano Isontina University Health Authority (Azienda Sanitaria Universitaria Giuliano Isontina – ASUGI) and covers Trieste and the nearby territory of Gorizia (catchment area: 370,000 inhabitants).

The eligible sample consisted of professionals from the Mental Health Department and the third sector working in synergy with the Department. Staff hired after March 2020 (first COVID-19 lockdown in Italy) were excluded from the study. The expected sample was 248 Mental Health Department staff and 174 third sector staff, respectively. Potential participants were invited to complete online an anonymous questionnaire on their views regarding the positive changes occurred in the MHS during the pandemic. The questionnaire was specifically developed for the purposes of the study by using a participatory approach (see assessment instrument section).

The study was coordinated by the Department of Psychology of the University of Campania “Luigi Vanvitelli”, Caserta, Italy and conducted in collaboration with the Department of Mental Health of the Giuliano Isontina University Health Authority (ASUGI), Trieste, Italy. Study protocol was approved by the Ethics Committee of the Department of Psychology of the University of Campania “Luigi Vanvitelli”, Caserta, Italy (authorization n. 1 of 2/2/2021) and acknowledged by the Office of Clinical and Epidemiological Studies, SC Research and Innovation, ASUGI, Trieste, Italy. The study was conducted in accordance with the Helsinki Declaration.

### Setting

The Trieste MHS network adheres to a no-restraint system of care and operates according to “open door” and maximum accessibility principles (non-selection of demands) [19]. It includes community mental health centers, a general hospital psychiatric unit, and residential facilities. The community mental health centers are open 24 h a day, 7 days a week and cover most care needs, including crisis management, mental disorder prevention, and pharmacological and rehabilitative interventions. The general hospital psychiatry unit is a six-bed ward located in the General Hospital of Trieste and serving the entire catchment area of the Trieste MHS. This inpatients unit is dedicated to voluntary and compulsory admissions of persons with acute psychiatric symptoms. It is mainly used for night emergencies and the duration of admissions is short (a few days): people are transferred as soon as possible from the psychiatric unit of the general hospital to the reference mental health center, where there are additional beds for the community management of acute psychiatric conditions. Residential care is provided by supported housing facilities managed by the third sector in collaboration with the Mental Health Department. The Mental Health Department collaborates with many social cooperatives promoting recovery, social inclusion, and employment programs. Individuals are supported in accessing social opportunities and in pursuing individualized rehabilitative plans, developed using a health budget approach [8, 27]. The Trieste MHS network is listed by the WHO as one of the most innovative and well-established community mental health systems [28, 29].

### Data Collection

The online data collection was conducted from February 15 through March 31, 2021. At the beginning of the data collection and twice during it, potential participants received an email from the employer inviting them to participate in an online study requesting their views regarding the transformations that occurred in the service during the first year of the COVID-19 pandemic. Those who accepted completed the Questionnaire on MHS Transformations during the COVID-19 pandemic—Staff version (QT-S). The research was also presented at the monthly team meeting by the Mental Health Department Director in March 2020. Participation was also solicited by word of mouth and WhatsApp by Mental Health Department coordinators and referents from cooperatives.

Participants completed the QT-S online using their personal devices (staff received a link to Google forms via invitation e-mails and WhatsApp messages). The possibility to stop the completion of QT-S and delete the answers already given was guaranteed by the online mode (simply close the Internet page without saving).

### Assessment Instrument

The QT-S is a self-reported assessment instrument exploring staff views on potential positive changes occurred in MHS during the pandemic. The QT-S development took place from September to December 2020 by a workgroup including researchers, clinicians, and coordinators of the main health sectors involved in the study. Initially, workgroup members individually examined six video interventions by staff presented at a webinar on the effects of COVID-19 pandemic on the MHS network of Trieste and Gorizia, held in September 2020 [6]. Using an ad hoc form, each participant extrapolated the prevailing themes from the video-interventions and rephrased the speakers' statements as multiple-choice items. This process led to a preliminary list of 232 items that were individually scored by the participants for clarity/appropriateness on a 10-point scale,—ranging from 1 “not at all” to 10 “completely”—and for inclusion/exclusion in the questionnaire. Items with a clarity/appropriateness score < 7 and/or suitable for inclusion by no more than 4/7 participants were eliminated (N = 160). The remaining 72 items were revised by the workgroup, and 25 redundant items were removed. The 47 items were re-evaluated for clarity/usefulness and inclusion/exclusion, and additional 17 items were excluded. The final 30 items were grouped on the following content basis: a) Acknowledgement of user capabilities (5 items); b) Awareness and value of teamwork (8 items); c) Flexibility and ability to reinvent the service (4 items); d) Maintenance and introduction of best practices (12 items); e) Acknowledgement of positive aspects in the pandemic experience (1 item). The rating scale included 6 levels, ranging from 1 “not really true” to 6 “really true.” In addition to the 30 items there was: an initial section of study aims and informed consent to participate and publish data; two additional open-ended items on respondent views of the most positive and most negative effects of COVID-19 pandemic on MHS; and, a section on the respondent’s main sociodemographic and professional characteristics.

### Statistical Analysis

Descriptive statistics were computed based on QT-S 30-items and the main socio-demographic and professional characteristics. Confirmatory Factorial Analysis was performed on the global sample to verify QT-S construct validity. Maximum likelihood estimation of the covariances was applied. Cronbach’s α values were computed to explore content validity of the confirmed factors. Multivariate Analysis of Variance and Bonferroni’s post-hoc were used to compare the mean scores of the five QT-S factors (dependent variables) with respect to main work settings (community mental health centres vs. social cooperatives vs residential facilities vs. general hospital psychiatric unit—independent variable). Statistical significance level was set at *p* < 0.05. Confirmatory Factorial Analysis was performed using the LISREL 8 Program [16]. Other tests were performed using the SPSS package, version 21 [13].

## Results

A total of 184 professionals completed the online questionnaire (participation rate: 43.6%). Participants had a mean age of 46.3 ± 10.5 (SD) years and most of them were females (61.4%), and highly educated (68.5%). Participants’ professional roles were as follows: psychiatrist 9.2%; psychologist 6.0%; nurse 31.0%; health care assistant 4.9%; educator/rehabilitator 23.8%; social cooperatives worker 16.3%; social worker 1.6%; administrative staff 3.3%; other/not specified: 3.7%. Participants worked in the mental health field for 13.8 ± 9.5 years on average. Of the 184 participating professionals, 48.4% worked in community mental health centers, 31.0% in social cooperatives, 7.6% in the general hospital psychiatric unit, and 6.5% worked in residential facilities (missing data 6.5%).

Ninety-two percent of participants felt it was “true/definitely true” (scores 5 and 6) that during the pandemic they had informed users on procedures to reduce individual risk of contagion, and 82.6% stated that they had taken greater care in the cleanliness of the environments (Table 1). Eighty-two percent stated that they had increased telephone contact with users, and 69.3% reported that staff revised individual treatment plans according to users’ new needs of care. Moreover, 58.6% of respondents stated that they had paid greater attention to users’ physical health, and 66.2% indicated that they had managed people in crisis as much as possible at home. Seventy-nine percent of staff learned to use digital technologies to work with public and private social institutions. Seventy-nine percent stated that the staff had been able to mediate between user needs and safe working procedures and 57.1% indicated that they had become more flexible regarding working alongside people during the pandemic. Moreover, 79.4% of respondents stated that the team had rediscovered the importance of gestures and habits, and 65.0% that they had gained strength among colleagues to face fear. Fifty-four percent of participants admitted that they had discovered personal resources in many users that they had not expected, and 35.5% acknowledged that persons with severe mental disorders had shown good adaptive skills in the pandemic. Overall, 59.6% of participants stated that they found some positives in the COVID-19 pandemic experience.

**Table 1:** Views of MHS staff on positive changes during the first year of the COVID-19 pandemic (N = 184)

QT-S items	Not really true Really true											
	1		2		3		4		5		6	
	N	%	N	%	N	%	N	%	N	%	N	%
**Acknowledgement of user capabilities**												
*, people with severe mental disorder demonstrated good adaptive skills	3	1.6	25	13.7	41	22.4	49	26.8	57	31.1	8	4.4
*, I discovered personal resources in users that I did not believe they had	7	3.8	9	4.9	21	11.5	46	25.3	64	35.2	35	19.2
*, users organized themselves into peer support groups	34	19.5	43	24.7	45	25.9	29	16.7	14	8.0	9	5.2
*, users showed that they were able to self-organize and find new solutions	12	6.7	23	12.8	33	18.4	57	31.8	41	22.9	13	7.3
*, I realized that there were users who know how to use digital technologies better than I did	29	16.4	27	15.3	24	13.6	36	20.3	30	16.9	31	17.5
**Awareness and value of teamwork**												
*, we realized the importance of simple gestures like drinking coffee together, shaking hands, hugging each other	6	3.3	6	3.3	10	5.4	16	8.7	43	23.4	103	56.0
*, there was more sharing of responsibilities within the team	10	5.5	16	8.7	26	14.2	46	25.1	54	29.5	31	16.9
*, more centrality was given to the meetings and to dialogue with the other person	6	3.3	14	7.8	35	19.4	57	31.7	43	23.9	25	13.9
*, I had more time to think about my work	15	8.2	22	12.1	31	17.0	37	20.3	46	25.3	31	17.0
*, we colleagues strengthened each other to face the fear	7	3.8	14	7.7	20	10.9	23	12.6	67	36.6	52	28.4
*, the sense of being part of a team strengthened	16	8.8	19	10.5	26	14.4	55	30.4	42	23.2	23	12.7
*, service meetings were an opportunity for group reflection on work practices	8	4.4	14	7.7	24	13.2	42	23.1	61	33.5	33	18.1
*, the guidelines of the Local Health Authority on how to work safely made us feel more reassured	26	14.3	28	15.4	40	22.0	43	23.6	33	18.1	12	6.6
**Flexibility and ability to reinvent the service**												
*, we became more flexible toward remaining close to users	7	3.8	12	6.6	20	11.0	39	21.4	53	29.1	51	28.0
*, we reinvented our way of working in line with government mandates	2	1.1	5	2.8	10	5.5	20	11.0	62	34.3	82	45.3
*, we were able to make organizational/operational changes very quickly	6	3.3	5	2.7	16	8.8	34	18.7	59	32.4	62	34.1
*, we mediated between user demands and the procedures needed to work safely	1	0.5	0	0	6	3.3	32	17.6	71	39.0	72	39.6
**Maintenance and introduction of best practices**												
*, we increased phone contact with users	4	2.2	4	2.2	9	5.0	15	8.4	55	30.7	92	51.4
*, we took more better care maintaining cleanliness in the work environment	2	1.1	6	3.3	12	6.5	12	6.5	58	31.5	94	51.1
*, we placed more importance on family members and the close relationships of users	4	2.2	11	6.1	27	15.0	43	23.9	60	33.3	35	19.4
*, we learned to use digital communication technologies to work with other public institutions and the third sector	1	0.5	2	1.1	11	6.0	25	13.7	56	30.8	87	47.8
*, we paid more attention to the physical health of users	5	2.8	4	2.2	16	8.8	50	27.6	66	36.5	40	22.1
*, we redefined the use of service spaces in a more rational way	5	2.8	10	5.6	15	8.4	36	20.1	51	28.5	62	34.6
*, we revised the users' programs according to their new needs	2	1.1	3	1.7	19	10.6	31	17.3	71	39.7	53	29.6
*, learned to use the PC and digital technologies better (e.g., video calling and conferencing platforms)	10	5.5	12	6.6	15	8.2	41	22.5	44	24.2	60	33.0
*, the CMHC remained the key point of referral for people with a fragile/absent family network	9	5.2	11	6.4	13	7.5	27	15.6	47	27.2	66	38.2
*, during hospitalization we guaranteed the contact of users with their families	2	1.2	11	6.7	15	9.1	29	17.7	57	34.8	50	30.5
*, we managed people in crisis as much as possible at home	4	2.3	5	2.9	17	9.9	32	18.6	57	33.1	57	33.1
*, we informed users about the pandemic and how to reduce individual risk of infection	0	0	1	0.5	5	2.7	9	4.9	62	34.1	105	57.7
**Acknowledgement of positive aspects in the pandemic experience**												
*, I found some positives in this experience	8	4.4	12	6.6	17	9.3	37	20.2	54	29.5	55	30.1

^*^During the pandemic

Confirmatory Factorial Analysis performed on the 30 QT-S items, confirmed the five-factor structure. The final model fit the data well, χ^2^(396) = 900.24, *p* < 0.001; non-normed fit index [NNFI] = 0.90; comparative fit index [CFI] = 0.91; root mean square error of approximation [RMSEA] = 0.087, 90% C.I. (0.080; 0.095); standardized root mean square residual [SRMR] = 0.086. All factor loadings were significant at the *p* < 0.001 level. Cronbach’s *α* values ranged from 0.68 to 0.83 (see Table 2 for details of the psychometric analyses).

**Table 2:** Confirmatory Factorial Analysis (CFA) on the 30 items of the QT-S (N = 184)

Items	Factor loadings				
	1	2	3	4	5
*, people with severe mental disorder demonstrated good adaptive skills	.42				
*, I discovered personal resources in users that I did not believe they had	.72				
*, users organized themselves into peer support groups	.60				
*, users showed that they were able to self-organize and find new solutions	.71				
*, I realized that there were users who know how to use digital technologies better than I did	.33				
*, we realized the importance of simple gestures like drinking coffee together, shaking hands, and hugging each other		.43			
*, there was more sharing of responsibilities within the team		.74			
*, more centrality was given to the meetings and to dialogue with the other person		.68			
*, I had more time to think about my work		.42			
*, we colleagues strengthened each other to face the fear		.64			
*, the sense of being part of a team strengthened		.83			
*, service meetings were an opportunity for group reflection on work practices		.70			
*, the guidelines of the Local Health Authority on how to work safely made us feel more reassured		.60			
*, we became more flexible toward remaining close to users			.70		
*, we reinvented our way of working in line with government mandates			.54		
*, we were able to make organizational/operational changes very quickly			.63		
*, we mediated between user demands and the procedures needed to work safely			.57		
*, we increased phone contact with users				.31	
*, we took more better care maintaining cleanliness in the work environment				.48	
*, we placed more importance on family members and the close relationships of users				.54	
*, we learned to use digital communication technologies to work with other public institutions and the third sector				.45	
*, we paid more attention to the physical health of users				.69	
*, we redefined the use of service spaces in a more rational way				.61	
*, we revised the users' programs according to their new needs				.72	
*, I learned to use the PC and digital technologies better (e.g., video calling and conferencing platforms)				.50	
*, the mental health center remained the key point of referral for people with a fragile/absent family network				.35	
*, during hospitalization we guaranteed the contact of users with their families				.32	
*, we managed people in crisis as much as possible at home				.34	
*, we informed users about the pandemic and how to reduce individual risk of infection				.41	
*, I found some positives in this experience					1
Cronbach’s *α*	.68	.83	.71	.79	-

^*^During the pandemic; Factor 1: Acknowledgement of user capabilities; factor 2: Awareness and value of teamwork; factors 3: Flexibility and ability to reinvent the service; factor 4: Maintenance and introduction of best practices; factor 5: Acknowledgement of positive aspects in the pandemic experience. The correlations among the five factors were all significant for p < .001 (standardized solution)

Multivariate Analysis of Variance showed significant differences between the facilities with respect to staff perception of maintenance/introduction of practices, service flexibility, and acknowledgement of users’ capacities (Wilks's λ = 0.72, F (10, 330) = 5.9, *p* < 0.0001). Bonferroni's post hoc highlighted a greater perception of changes in practices among community mental health centers staff vs. general hospital psychiatric unit and residential facilities staff; and, a greater perception of service flexibility among social cooperatives staff vs. general hospital psychiatric unit and residential facilities staff. The analysis also showed a tendency for greater acknowledgement of users' capabilities among community mental health centers staff vs. social cooperatives staff (*p* = 0.56). No other significant difference between facilities was detected (Table 3).

**Table 3:** View**s** of mental health services staff on positive changes during the pandemic: differences between work settings

	Participants’ workplaces			MANOVA
QT-S factors	Residential facilities and general hospital psychiatric unit (N = 26)	Community mental health centers (N = 89)	Social cooperatives (N = 57)	F (2, 172)
	mean ± sd	mean ± sd	mean ± sd	
Acknowledgement of user capabilities	3.5 ± 0.9	3.8 ± 0.9	3.5 ± 0.9	3.57, < .03
Awareness and value of teamwork	3.8 ± 0.7	4.2 ± 0.9	4.3 ± 1.0	2.08, NS
Flexibility and ability to reinvent the service	4.5 ± 0.8^b^	4.8 ± 0.9^a, b^	5.1 ± 0.8^a^	5.90, < .003
Maintenance and introduction of best practices	4.5 ± 0.5^b^	5.0 ± 0.6^a^	4.7 ± 0.6^a, b^	6.39, < .002
Acknowledgement of positive aspects in the pandemic experience	4.3 ± 1.4	4.5 ± 1.3	4.6 ± 1.5	0.65, NS

*12 missing data excluded from Multivariate Analysis of Variance (MANOVA); Bonferroni post-hoc comparisons: a > b; NS = non-significant p value

## Discussion

### Interpretation of the Results

The results of this study indicated that after one year of the pandemic, most MHS staff felt they had been able to change practices and reinvent the work organization, reevaluating teamwork and recognizing capabilities to users. The study also revealed that perceptions of positive changes were greater among professionals working in the community than among those working in hospital/residential facilities.

Most participants stated that they had found some positives in the pandemic experience. This belief may reflect the high percentage of “true/completely true” responses to most items, which, in turn, could be an indirect indicator of the service resiliency. It is likely that the one-year assessment gave enough time for people to use their inherent capacities to bounce back. It should be noted that positive changes involve several aspects of MHS functioning, such as revision of therapeutic plans in light of new needs for care, greater attention to users’ physical health, and increased home-based crisis management. In line with other studies [4, 15, 26], most participants reported an increased use of digital technology. The use of video-communication platforms not only reduced travel-related workloads but also led to devising innovative treatment packages. In particular, since the early pandemic phase, a platform for help care, entertainment and training was developed to integrate into the healthcare programs. The “Platform for Inclusion” enhanced home activities, facilitated the acquisition of skills and new educational tools by the users, and reduced loneliness [6]. Effectiveness studies are needed to assess the long-term impact and feasibility of digital resources on MHS in the COVID-19 pandemic era [10].

Fifty-seven percent of the participants indicated that they had become more flexible regarding working alongside people during the pandemic, a percentage higher than that reported by D'Avanzo et al. [4] in the early COVID-19 pandemic, where 32% of MHS staff pointed out greater work flexibility. In line with previous research data [15], most professionals revalued the teamwork and relied upon colleagues’ support to cope with contagion fear. Interestingly, 54.4% of participants were convinced that they discovered personal resources in users that they did not expect to find, and 35.5% acknowledged good coping skills to people with severe mental disorders. As hypothesized by some authors [9, 26], it might be that the reduced social pressure facilitated the adaptation of users with severe mental disorders to the pandemic. Another explanation could be that the pandemic reduced users’ social opportunities within the MHS. This may have led some to revalue external social relationships that, in turn, reinforced coping skills. Regardless of the motivation, the staff acknowledgement of users’ capacities is encouraging, since this can positively influence stigma in MHS [18, 25] and facilitate users’ empowerment. Ad hoc studies are needed to estimate the long-term impact of the pandemic on the staff-user relationships and the recovery processes.

The study found significant differences in positive changes between facilities. Specifically, compared to residential and hospital facilities staff, professionals in community services had greater perceptions of positive changes in the practices and valued the services as more capable of adapting to users’ new needs. These differences, in line with previous findings [4, 15], highlight the greater manageability of the pandemic in community MHS, which is partially related to long-term collaboration with the third sector and the user and family associations [7]. The lower perception of positive changes by the general hospital psychiatric unit and residential facilities staff could also be related to users’ clinical conditions and facilities’ characteristics requiring stricter rules to limit contagions [5].

Overall, the encouraging results of this study can be interpreted within the framework of Lazarus and Folkman’s [17] transactional model, which postulates that the adaptation to an event is a process based on primary and secondary cognitive appraisal. Regarding the impact of COVID-19 pandemic on MHS, primary appraisal referred to what the pandemic was, as documented by the early-phase studies. Secondary appraisal concerned the emotional and problem-oriented strategies to cope with the pandemic in the long-term. Internal factors, such as individual attitudes and commitment to work, and external factors, such as MHS approach and team cohesion, may have influenced the adaptation process. This, in turn, may have led professionals to recognize several positive changes in their work during the pandemic, despite the difficulties they encountered [2, 22].

### Strength and Limitations of the Study

This is the first study conducted in Italy that systematically explored staff views on the positive transformations occurring in MHS during the first year of the pandemic. The multidisciplinary working group and the participatory approach facilitated the development of an instrument, which was well accepted by participants (low percentage of missing responses) and encompassed different perspectives. The fact that the QT-S is rapid to fill in and has good psychometric properties will facilitate the study replication in other settings as well as data comparison. Moreover, the fact that the QT-S items can be grouped into five higher-order dimensions makes it easier to interpret the results.

The survey was conducted in a single MHS network whose organizational model, largely adopted throughout the Friuli Venezia Giulia region [3], is strongly community-oriented. Therefore, the results of this study may not be fully generalizable to services with different patterns of care. Moreover, a total of 43.6% of eligible participants completed the questionnaire. It cannot be excluded that non-participants had a more negative perception of the impact of COVID-19 pandemic on the service. Therefore, the results may overestimate the positive changes in the MHS. In addition, the research investigated staff *perceptions* of positive changes and it did not explore whether such perceptions reflected real changes in the service. Nor can it be ruled out that responses were influenced by social desirability, despite the anonymity in data collection. Finally, the study examines changes from the view of staff, not considering the perspectives of users and their families. Some of these limitations will be addressed in further studies, which are currently in the planning stages.

## Conclusions

This survey sought to examine the pandemic with a new approach, i.e., looking at the positive changes rather than the negative impact it produced. The study provided a snapshot of a community-oriented MHS network that looked back and gave an all-around positive rating of its performance in the first year of the COVID-19. The results of this study highlight that, in a community-oriented MHS, the pandemic also represented—and continues to represent—an opportunity to change practices and the relationships among professionals and between professionals and users. We hoped that these data will contribute to the understanding of the impact of COVID-19 on MHS and to the design of community-oriented and person-centered services in the pandemic era [10, 28].

## Data Availability

The data that support the findings of this study are available from the corresponding author upon reasonable request, which must include a protocol and statistical analysis plan and not be in conflict with our publication plan.
